# Bayesian inference captures metabolite–bacteria interactions in a microbial community

**DOI:** 10.1093/ismeco/ycag066

**Published:** 2026-03-21

**Authors:** Jack Jansma, Pietro Landi, Cang Hui

**Affiliations:** Biodiversity Informatics Unit, Department of Mathematical Sciences, Stellenbosch University, Stellenbosch 7600, Western cape, South Africa; Biodiversity Informatics Unit, Department of Mathematical Sciences, Stellenbosch University, Stellenbosch 7600, Western cape, South Africa; National Institute for Theoretical and Computational Sciences, Stellenbosch 7600, Western cape, South Africa; Biodiversity Informatics Unit, Department of Mathematical Sciences, Stellenbosch University, Stellenbosch 7600, Western cape, South Africa; National Institute for Theoretical and Computational Sciences, Stellenbosch 7600, Western cape, South Africa; African Institute for Mathematical Sciences, Cape Town 7100, Western cape, South Africa

**Keywords:** Bayesian inference, microbial community, parameter estimation, mathematical modelling, metabolite–bacteria network, microbiota-targeted interventions, gut microbiota, dynamic network model

## Abstract

Macro-ecosystems, including the human gut, host a vast and diverse set of microbes that indirectly interact with each other through consuming and producing metabolites. Disruptions in this microbial network can affect macro-ecosystem functioning and, in the human gut, contribute to the onset and progression of various disorders, including diabetes, rheumatoid arthritis, and Parkinson’s disease. A theoretical foundation for understanding the intricate and dynamic interactions between microbes and metabolites is essential for developing microbiota-targeted interventions to improve macro-ecosystem functioning and health. To this end, a precise mathematical framework is crucial to capture and quantify the complex dynamics of the microbial system. Here, we develop a dynamic network model of coupled ordinary differential equations and present a computational workflow that integrates a generative model with Bayesian inference for model identification. Our approach infers interaction rates, quantifying metabolite consumption and production from simulated time-series data within a Bayesian framework, incorporating prior knowledge and uncertainty quantification. We show that our approach is accurate and reliable in communities of various sizes, sparsity, and with different levels of observational noise. This workflow enables *in silico* predictions of system behaviour under perturbations and offers a robust method to integrate high-dimensional biological data with dynamic network models. By refining our understanding of microbial dynamics, this framework is capable of assessing microbiota-targeted interventions and their potential to improve the health of the macro-ecosystem.

## Introduction

Microbial communities are ubiquitous in nature and play a vital role in all macro-ecosystems such as the soil, ocean, and human gut [1]. Disruption of the delicate balance of microbial communities can be detrimental to the macro-ecosystem. For example, disruption of the soil microbiota alters the regulation of carbon and nutrient cycling, directly impacting macro-ecosystem resilience [1]. Alterations in the composition of the gut microbiota have been implicated in the onset and progression of various disorders including diabetes, rheumatoid arthritis, and Parkinson’s disease [2, 3]. Accordingly, restoration of disturbed microbial communities is a key aspect in the conservation of macro-ecosystems and in the prevention and treatment of disease. However, a lack of theoretical and computational frameworks of the underlying ecological structure hampers our understanding of microbial communities [4] and subsequently our understanding of the role of these communities in the macro-ecosystem. Developing a theoretical model of microbial communities can support the design of field or wet lab experiments and guide the development of novel interventions that can ultimately improve the health of the host or macro-ecosystems.

Microbial communities are characterized by their composition and abundance but more precisely by their interactions [5]. The majority of these interactions, however, are indirect and mediated via the metabolic environment, specifically via microbial consumption and secretion of metabolites [6]. Therefore, the functioning of a microbial community can be described realistically as the network of these indirect metabolic interactions. Indeed, metabolic concentrations and microbial abundances can be measured experimentally in a community with established methods including metabolomics, sequencing, or flow cytometry techniques [7, 8]. However, experimentally measuring the direction and strength of these metabolic interactions is not as straightforward, especially when the interactions are numerous and sporadic, making the quantification of metabolic networks challenging. Most studies use computational methods to infer and quantify microbe–metabolite interactions [9, 10]. For instance, correlations between relative abundances of bacteria across time points (and locations) can serve as edge weights in constructing the association network. When combined with bipartite host-microbe networks, these correlation-based methods have revealed distinct nested structures that define the gut microbiota of healthy humans [11]. While correlation-based methods are useful for identifying potential microbe–metabolite interactions, they mainly rely on cross-sectional data and thus overlook the dynamic and context-dependent nature of microbe–metabolite interactions [12].

Adopting a dynamic model not only offers a better representation of the microbiota but also paves the way for characterizing the stability and resilience of the microbial system and potentially enhances the predictability of microbiota-targeted interventions through scenario simulations. However, longitudinal microbiota studies are costly, limiting the duration and frequency of data collection. For instance, longitudinal sample collection of stool usually takes place in the order of days, whereas the changes within the gut microbiota in response to a perturbation are in the order of hours [13]. Consequently, estimating the frequency and duration of the sampling protocol for longitudinal studies *a priori* becomes essential [13]. Moreover, investigating the dynamics of hard-to-reach microbial communities, such as the small intestinal microbiota, is experimentally challenging [14]. Employing an *in silico* approach, utilizing generative models and statistical inference, can circumvent some of these limitations and better guide the design of field and wet lab experiments, so long as the model itself captures the key dynamic processes of the system.

Dynamical processes in microbial communities are well described using ordinary differential equations (ODEs), and parameter inference is well established using frequentist methods such as least squares [15]. However, these methods usually require large amounts of data and specific assumptions about the residual distribution to provide reliable estimates. When data are sparse, as is common in high-dimensional, nonlinear systems such as in microbiota dynamics, least-squares estimation can become unstable and prone to overfitting, and may underestimate uncertainty, leading to misleading point estimates. In addition, it offers no formal way to incorporate prior knowledge [16]. In contrast, Bayesian inference naturally accounts for uncertainty in the data through likelihood functions and incorporates prior information, yielding posterior distributions from which credible intervals and parameter correlations can be derived. It also enables direct assessment of whether the inferred model reproduces observed data patterns. Moreover, Bayesian methods are well suited for nonlinear models and, by combining priors with the likelihood, can stabilize parameter estimation and address issues of identifiability (i.e. different combinations of parameters can explain the data equally well, making it difficult to distinguish among them) [17].

Here, we develop a bacteria-metabolite model using a set of ODEs, describing the consumption and excretion of metabolites by bacteria in a community. Using simulated data from this generative model, we employ a Bayesian inference framework to quantify bacterial–metabolite interactions in bacterial communities of various sizes and sparsity. Our *in silico* approach allows us to assess parameter recovery and robustness of our framework in a controlled environment without the interference of noise and complexity as is observed in a real-world context. Using Bayesian inference to address model identifiability further allows us to inform the design of longitudinal experiments for optimized sampling strategies. The combination of a generative mathematical model as a hypothesized ground truth with a Bayesian statistical approach for model testing and inference, therefore, contributes to our fundamental understanding of microbial community functioning.

## Material and methods

### Model construction

Our model is defined by a set of coupled ODEs that describe the dynamics of bacteria and metabolites in a community [18]. Let ${M}_i$ depict the concentration of metabolite $i$ and ${B}_j$ the abundance of bacterium $j$ in a microbial community, where all bacteria solely interact indirectly via consuming and excreting metabolites in the shared metabolic environment (denoted by vector $\boldsymbol{M}=\left\{{M}_1,{M}_2,\dots \right\}$). After considering the death rate ${d}_j$ of bacterium $j$ in a finite environment with carrying capacity $k$, we can consider the per-capita growth rate, ${g}_j\left(\boldsymbol{M}\right)$, that depends on the uptake and assimilation of multiple metabolites.

Consider, for example, the simplest form, where assimilated metabolites are substitutable and additively contribute to the per-capita growth rate, ${g}_j\left(\boldsymbol{M}\right)={c}_0\sum_{i\in \left\{M\right\}}{c}_{i,j}^{+}{M}_i$, where ${c}_0$ depicts the metabolite conversion coefficient and describes how much of the total consumed metabolites is converted into population growth, and ${c}_{i,j}$ depicts the consumption (${c}_{i,j}>0$) or excretion (${c}_{i,j}<0$) rate of metabolite $i$ by bacterium $j$, with ${c}_{i,j}^{+}=\max \left({c}_{i,j},0\right)$ representing the fact that only consumed metabolites support cellular processes and contribute to bacterial population growth. Metabolites excreted by a bacterial species into the environment do not affect its own population growth but can be consumed by other species, creating cross-feeding interactions. This yields the following population dynamics of the bacteria:


1 \begin{eqnarray*} \frac{{\mathrm{d}B}_j}{\mathrm{d}t}={B}_j\left({g}_j\left(\boldsymbol{M}\right)-{d}_j\right)\left(k-\sum_{l\epsilon \left\{B\right\}}{B}_l\right), \end{eqnarray*}

where $\left\{B\right\}$ is the set of bacteria species. We assume that the consumption of metabolites is sufficiently fast to deem the term for metabolic outflow or degradation unnecessary. The dynamics of the metabolites are therefore defined as:


2 \begin{eqnarray*} \frac{d{M}_i}{dt}={s}_i\left[t\right]-{M}_i\left(\sum_{j\in \left\{B\right\}}{c}_{i,j}{B}_j\right), \end{eqnarray*}

where ${s}_i\left[t\right]$ depicts the supply rate of metabolite $i$ at time $t$, with ${s}_i\left[t\right]=0$ representing a closed system where the initial metabolite concentration ${M}_i\left[0\right]$ can only increase from bacterial secretion (${c}_{i,j}<0$) or decline from bacterial consumption (${c}_{i,j}>0$). Note, the system becomes open if ${s}_i\left[t\right]\ne 0$, which can reflect the manipulation of the metabolic environment.

For the purpose of this study, we fixed the death rate, ${d}_j$, at 0.1. This value was derived from a series of experiments using dilution as a proxy for death, conducted with a three-species bacterial community that exhibited coexistence at a dilution rate of 0.1 [19]. We fixed the metabolite conversion coefficient, ${c}_0$, of each bacterium at 0.1, based on a dynamic energy allocation model parameterized using experimental data from *Escherichia coli* [20]. We fixed the carrying capacity at 14, which is approximated and scaled from the total number of bacterial cells (14 billion per millilitre) obtained in a wet lab experiment [21]. We also fixed ${s}_i\left[t\right]=0$ in Eq. (2) to represent the closed system in this wet lab experiment. Consequently, the focus below is to estimate the interaction coefficients, ${c}_{i,j}$, using count and concentration readings of ${B}_j$ and ${M}_i$, respectively, from snapshots of the microbiota at different time points.

To generate synthetic data of modelled microbiota dynamics, we randomly assigned interaction coefficients, ${c}_{i,j}$, and initial conditions $\left({M}_i\left[0\right],{B}_j\left[0\right]\right)$and solved the model using the Runge–Kutta 4(5) solver (ode45) in the R package deSolve. Let the observations of the quantity of bacterial or metabolic species, $l$, at timepoint, $t$, in replicate, $r$, be denoted as, ${y}_{\mathrm{obs}}=\left\{{y}_{r,t,l}\right\}$. Note, the observed value ${y}_{r,t,l}$ and the predicted value ${\mu}_{r,t,l}$ from the model (Eqs 1 and 2, representing the assumed microbiota dynamics) are both non-negative, and they can be different due to the observation error ${\varepsilon}_{r,t,l}$ defined on the logarithmic scale,


3 \begin{eqnarray*} {\varepsilon}_{r,t,l}=\log \left({y}_{r,t,l}\right)-\log \left({\mu}_{r,t,l}\right), \end{eqnarray*}

where, in practice, a small positive constant $\delta$ close to zero is added in the logarithm to avoid a zero quantity. Observation errors can arise due to sample handling and measurement error in real-world experiments. The observation error is assumed to follow a normal distribution with zero mean and standard deviation ${\sigma}_{\mathrm{err}}$. Consequently, the simulated observations can be generated from a log-normal distribution with the predicted value as the mean:


4 \begin{eqnarray*} {y}_{r,t,l}^{\mathrm{obs}}\sim \mathrm{LogNormal}\left(\log \left({\mu}_{r,t,l}\right),{\sigma}_{\mathrm{err}}\right). \end{eqnarray*}

First, we generated the dynamics of a small community, without observation error (${\sigma}_{\mathrm{err}}=0$ and thus ${y}_{r,t,l}^{\mathrm{obs}}={\mu}_{r,t,l}$), consisting of three bacteria and three produced and seven consumed metabolites, with their initial values randomly assigned from $\mathrm{Uniform}\left(0.05,0.2\right)$, $\mathrm{Uniform}\left({e}^{-3},{e}^{-2}\right)$ and $\mathrm{Uniform}\left(0.5,1\right)$, respectively, while the interaction coefficients of the produced metabolites (${c}_{i,j}^{\mathrm{Prod}}<0$) and the consumed metabolites (${c}_{i,j}^{\mathrm{Cons}}>0$) were randomly obtained from $\mathrm{Uniform}\left(-1,0\right)$ and $\mathrm{Uniform}\left(0,1\right)$, respectively. For each set of randomly assigned interaction coefficients, we generated six independent replicates using different initial conditions. The generated dynamics (${\mu}_{r,t,l}$) were observed at four evenly spaced timepoints between $t=0$ and $t=4$. Second, to assess the effect of observation error, we computed the observed value ${y}_{r,t,l}^{\mathrm{obs}}$ in Eq. (4) with the error standard deviation, ${\sigma}_{\mathrm{err}}$, independently drawn from $\mathrm{Uniform}\left(0.1,0.5\right)$ for metabolite concentrations and from $\mathrm{Uniform}\left(0.01,0.05\right)$ for bacterial densities, for each actual snapshot value, ${\mu}_{r,t,l}$, of the generated dynamics. Third, to assess the effect of irregular sampling, we observed the generated dynamics at 4 and 25 randomly generated timepoints between $t=0$ and $t=4$ with added observation error.

The workflow described above is implemented in R and is accessible via the GitHub link found in the Data Availability statement. Briefly, the R-script implements the generative model based on a system of ODEs and the Bayesian inference framework (see below), including values for all parameter values used in the generative model and the corresponding estimated interaction coefficients. Here, the user is able to specify the model structure (e.g. the growth function of microbiota) and generate data points for a specific system.

### Bayesian inference

In the Bayesian inference framework, the posterior distribution, $\mathrm{P}\left({c}_{i,j},{\sigma}_{\mathrm{res}}|{y}_{\mathrm{obs}}\right)$, of interaction coefficients, ${c}_{i,j}$, and residual standard deviation, ${\sigma}_{\mathrm{res}}$, is described by:


5 \begin{eqnarray*} \mathrm{P}\left({c}_{i,j},{\sigma}_{ar}|{y}_{\mathrm{obs}}\right)\propto \prod_{r=1}^R\prod_{t=1}^T\prod_{l=1}^L\mathrm{P}\Big({y}_{r,t,l}^{\mathrm{obs}}\Big|{\mu}_{r,t,l},{\sigma}_{\mathrm{res}}\Big)\mathrm{P}\left({c}_{i,j}\right)\mathrm{P}\left({\sigma}_{\mathrm{res}}\right), \end{eqnarray*}

where $\mathrm{P}\Big({y}_{r,t,l}^{\mathrm{obs}}|{\mu}_{r,t,l},{\sigma}_{\mathrm{res}}\Big)$ is the likelihood of observing ${y}_{r,t,l}^{\mathrm{obs}}$ given the model prediction ${\mu}_{r,t,l}$ and residual standard deviation, ${\sigma}_{\mathrm{res}}$; $\mathrm{P}\left({c}_{i,j}\right)$ and $\mathrm{P}\left({\sigma}_{\mathrm{res}}\right)$ depict the priors. To assess whether the Bayesian inference framework could correctly estimate the assigned interaction coefficients from simulated observations, we specified weakly informative priors to regularize estimation while retaining flexibility. Interaction coefficients were assigned according to their metabolite category: $\mathrm{P}\left({c}_{i,j}^{\mathrm{Prod}}\right)\sim \mathrm{Uniform}\left(-1,0\right)$ for produced metabolites and $\mathrm{P}\left({c}_{i,j}^{\mathrm{Cons}}\right)\sim \mathrm{Uniform}\left(0,1\right)$ for consumed metabolites. The prior for the residual standard deviation was specified as $\mathrm{P}\left({\sigma}_{\mathrm{res}}\right)\sim \mathrm{Normal}\left(0,0.2\right)$.

Bayesian inference was performed using Hamiltonian Monte Carlo with the No-U-Turn Sampler (NUTS), as implemented in the R package Rstan [22]. For simulated data without observation errors, inference was conducted with four chains of 2000 iterations, discarding the first 1000 iterations as warm-up. To reduce computational costs for all other simulated data with randomly assigned observation error, as well as for larger and sparser communities, two chains of 1000 iterations were run, with 500 warm-up iterations discarded. To improve sampling performance in the presence of complex posterior geometries, we set the target acceptance probability (adapt_delta) to 0.99, thereby reducing the risk of divergent transitions.

The quality of inference was evaluated through standard convergence diagnostics and posterior predictive checks. Convergence was assessed with the potential scale reduction statistic (Rhat), while sampling efficiency was quantified as the effective sample size (n_eff). Posterior predictive checks were performed by comparing the input observations with simulated observations obtained by solving the ODEs using the integrate_ode_rk45, as implemented in the R package Rstan, with interaction coefficients drawn from the posterior. Model accuracy was further assessed by calculating the mean absolute error (MAE) between the assigned interaction coefficients and their posterior means, as well as by calculating the coverage probability, which gives the proportion of assigned interaction coefficients that fall within the 95% credible intervals of the posterior distribution. The effect of zero inflation on the accuracy and uncertainty was assessed by calculating Pearson’s correlation coefficient between the posterior mean and the width of the 95% credible interval with the corresponding product of the metabolite and bacterial abundances, averaged across time.

The Bayesian inference framework described above is implemented in Rstan [22], which can be accessed via the GitHub link provided in the Data Availability statement. In short, the R-script allows the users to specify alternative model structures (formulated as a system of ODEs) and to incorporate experimentally obtained observations from real-world biological systems as sole input, instead of simulated datasets, for inferring interaction coefficients.

## Results

### Accurate and reliable estimation of interaction coefficients

**Figure 1:**
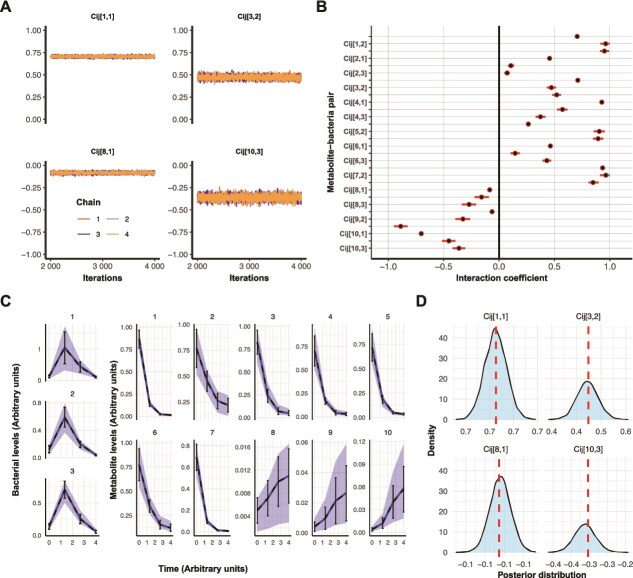
Simulation-based calibration accurately estimates interaction coefficients in a small bacterial community. (A) Trace plots of the posterior samples for four representative metabolite-bacteria interaction coefficients across four independent Hamilton Monte Carlo–No-U-Turn sampler (NUTS) chains. Each panel shows sampled parameter values over iterations. (B) Posterior distributions of all interaction coefficients, ${c}_{i,j}$. Posterior means are depicted as dots, with 95% credible intervals indicated by bars. (C) Posterior predictive check. The mean and standard deviation of four generated observations, obtained by numerically solving the set of ODEs using the assigned initial conditions and interaction coefficients, are shown as dotted lines and error bars. The mean and 95% credible intervals of generated observations, using draws from the estimated posterior distributions, are shown as a solid line and a belt. (D) Four representative comparisons of assigned interaction coefficients (dotted lines) with the corresponding posterior distributions.

For communities without observation errors, model diagnostics of the Bayesian inference framework indicated proper chain mixing (Fig. 1A), no divergent transitions, with all Rhat values below 1.002 and all n_eff above 5500, collectively indicating sampler convergence and reliable parameter estimation across all model parameters (Fig. S1A and B). Inspecting the posterior distributions showed that the credible interval is small and that the means are spread out across the full range of the assigned priors (Fig. 1B). Posterior predictive checks showed that solving the set of ODEs with interaction coefficients drawn from the posterior distributions produced predictions consistent with the observations (Fig. 1C), with posterior means overlapping well with the assigned interaction coefficients (Fig. 1D). Overall, Bayesian inference showed robust inferential performance and high parameter precision, underscoring methodological accuracy.

**Figure 2:**
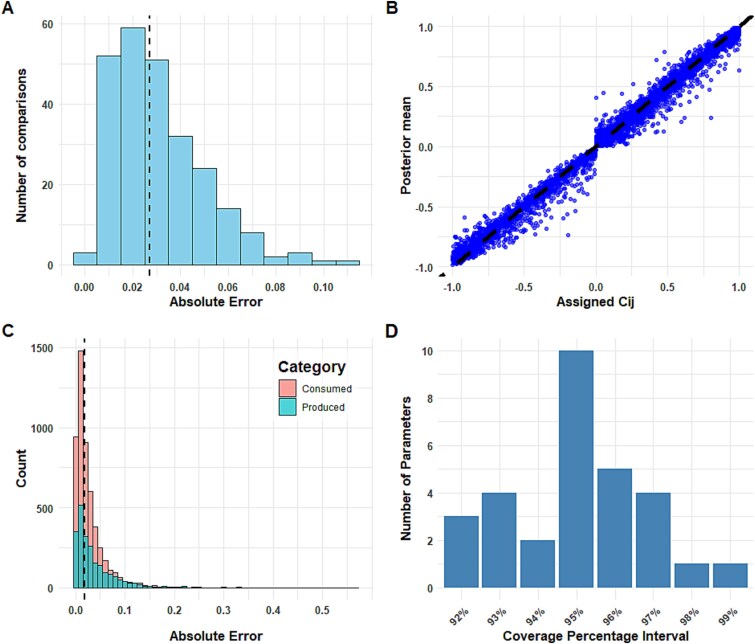
Independent simulations show reliable estimation of interaction coefficients in a small bacterial community. **(**A) Mean absolute differences between assigned interaction coefficients and corresponding posterior means across 250 simulations. The median is shown as the dotted line. (B) 7500 comparisons of assigned versus estimated parameters across 250 simulations. The *x* axis represents the assigned interaction coefficients (${c}_{i,j}$), and the *y* axis depicts the corresponding posterior mean. The dotted line indicates the 1:1 relationship expected if posterior means matched the assigned values exactly. (C) Absolute differences between assigned interaction coefficients and corresponding posterior means, plotted across 7500 parameters across 250 simulations. Interaction coefficients for produced and consumed metabolites are shown as separate categories. The median is shown as the dotted line. (D) Distribution of the percentage of assigned interaction coefficients that fall within the 95% credible interval of the posterior distribution across 250 simulations.

We evaluated the reliability of our inference approach by comparing the assigned interaction coefficients with the corresponding posterior mean across 250 independent simulations. The median of the MAE was 0.027, with 75% of simulations below 0.04, indicating consistent recovery of interaction coefficients (Fig. 2A). Next, we evaluated the accuracy by comparing every assigned interaction coefficient, ${c}_{i,j}$, with the corresponding posterior mean for all simulations, resulting in a total of 7500 comparisons (Fig. 2B). The median of these variable-specific comparisons was 0.018, with 75% of simulations below 0.04, with the maximum error obtained being 0.565 (Fig. 2C). Multiple parameter combinations can result in identical or nearly identical trajectories, particularly under finite noise and partial observations. Consequently, our nonlinear dynamical system is not guaranteed to be structurally identifiable as observation noise renders inference stochastic rather than deterministic. We therefore assessed the practical identifiability of our systems by calculating the coverage probability, representing the proportion of assigned interaction coefficients that fell within the 95% credible interval of their corresponding posterior distributions, indicating high reliability of our inference approach (Fig. 2D). Lastly, we evaluated the effect of zero-inflation on the accuracy of our approach since datasets obtained from large scale microbiota experiments often contain a high number of zeros. Examining the correlation between the product of metabolite and bacterial abundances averaged across time with the posterior mean (Fig. S2A) and the width of the 95% credible interval (Fig. S2B) indicates that our framework maintains accuracy, although with increased uncertainty under zero inflation conditions.

### Reduced uncertainty with increased number of observations

**Figure 3:**
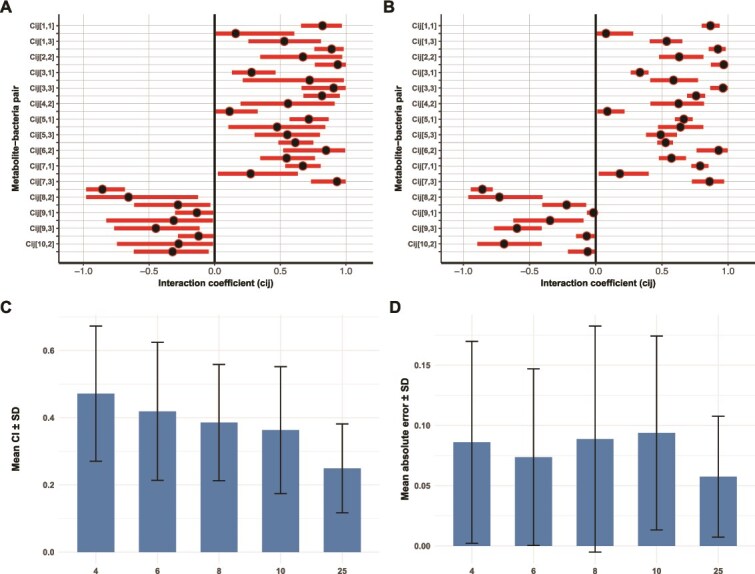
Estimation uncertainty increases with observation error but decreases with more observations. (A) Posterior distributions of all interaction coefficients (${c}_{i,j}$) in the small bacterial community using four observations with observation error added. Posterior means are shown as dots and 95% credible intervals as bars. (B) Posterior distributions of all interaction coefficients in the same community using 25 observations with observation error added. Posterior means are shown as dots and 95% credible intervals as bars. (C) Mean and standard deviation (SD) of 95% credible intervals (CI) across posterior distributions obtained from parameter estimation with varying numbers of observations in the small bacterial community with added observation error. (D) Mean and SD of the absolute error between assigned interaction coefficients and posterior means obtained from parameter estimation with varying numbers of observations in the small bacterial community with added observation error.

The addition of observation errors did not negatively affect the diagnostics of the Bayesian inference framework (Fig. S3A and B), although more uncertainty was found as indicated by broader posterior distributions (Fig. 3A), which was also reflected in the posterior predictive check (Fig. S3C). A straightforward way to increase the accuracy of parameter estimation is to increase the number of observations. We tested this hypothesis by increasing the number of observations of the small bacterial communities under random observation errors from 4 observations to 6, 8, 10, and 25, evenly spaced across the same temporal range. Model diagnostics was not affected by increased observations (Figs S4A and B and S5A and B), but the credible interval of the posterior distributions was reduced (Fig. 3B), with the smallest credible interval obtained using the largest number of observations (Fig. 3C). Furthermore, a posterior predictive check indicated accurate recovery of the observed data (Figs S4C and S5C). Although the MAE between the posterior means and the assigned interaction coefficients decreased slightly with 6 observations, it slightly increased with 8 and 10 observations, possibly due to minor discrepancies in numerical integrations at these intermediate time points. In contrast, using 25 observations consistently produced the lowest MAE (Fig. 3D). While increasing the number of observations reduced the uncertainty of parameter estimates, gains in accuracy plateaued beyond a certain point, with additional observations offering little improvement. Lastly, we assessed if irregular sampling, as is common during longitudinal studies, affected the accuracy and uncertainty of inference. To this end, we randomly and unevenly spaced 4 and 25 observations, including observation error, obtained from the small bacterial community across the same temporal range as before. Model diagnostics (Fig. S6A, B, D, and  F), uncertainty, and accuracy (Fig. S6G and H) were not affected, indicating the robustness of our inference method to irregular sampling. However, the bacterial and metabolite dynamics obtained from four irregular-spaced observations (Fig. S6C) were different from the dynamics obtained from 4 regular-spaced (Fig. 1C) and 25 irregular-spaced observations (Fig. S6E), highlighting the need for a higher number of timepoints and careful consideration of the sampling protocol.

### Accuracy declines with community size

**Figure 4:**
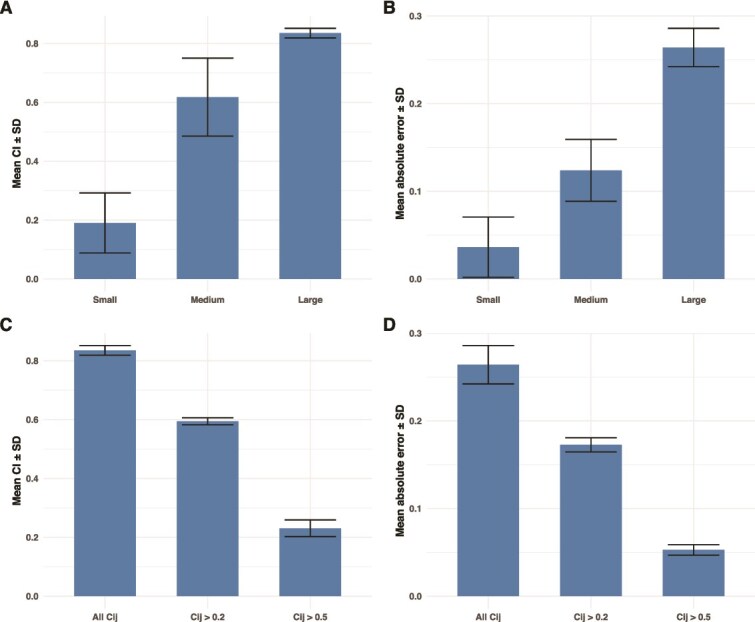
Estimation uncertainty and accuracy vary with community size and network sparsity. **(**A) Mean and standard deviation (SD) of the 95% credible intervals (CI) across posterior distributions obtained from parameter estimation using four observations for three community sizes (small: 3 bacteria and 10 metabolites; medium: 5 bacteria and 14 metabolites; large: 10 bacteria and 20 metabolites). (B) Mean and standard deviation (SD) of the absolute error between assigned interaction coefficients and posterior means for the same three community sizes. (C) Mean and SD of the 95% CI across posterior distributions for the large community (10 bacteria and 20 metabolites) under three sparsity conditions: All interaction coefficients included (all ${c}_{i,j}$), coefficients below 0.2 removed (${c}_{i,j}$ > 0.2), and coefficients below 0.5 removed (${c}_{i,j}$ > 0.5). (D) Mean and SD of the absolute error between assigned interaction coefficients and posterior means for the large community under the same sparsity conditions as in (C).

We only considered a small bacterial system with three bacteria and 10 metabolites, based on a synthetic community studied in wet lab experiments [21]. However, many synthetic communities contain more species and metabolites [23], and naturally occurring communities can be even larger and more complex [24]. To assess the feasibility and accuracy of our approach in larger communities, we applied Bayesian inference to 10 medium-sized communities consisting of 5 bacteria and 14 metabolites, and to 5 large communities consisting of 10 bacteria and 20 metabolites. Model diagnostics for both community sizes indicated no divergent transitions, with Rhat values close to one and large n_eff (Figs S7A and B and S8A and B). Comparisons of posterior distributions (Figs S7C and S8C), posterior predictive checks (Figs S7D and S8D), and comparison of the assigned interaction coefficients with corresponding posterior means (Figs S7E and S8E) showed consistent patterns across communities. Specifically, as community size increased, posterior means for consumed metabolites shifted towards 0, those for produced metabolites shifted towards −0.5, and the posteriors became flatter, indicating reduced predictive power. Furthermore, the credible intervals of the posterior distributions (Fig. 4A) and the MAE between the assigned interaction coefficients and corresponding posterior means (Fig. 4B), both increased with community size, indicating reduced accuracy and reliability of Bayesian inference in larger communities.

### Imposing sparsity of the interaction matrix improves estimation

In the analyses above, the number and combinations of parameters to be estimated by our Bayesian inference framework increased substantially with increasing community size. As a result, the accuracy and reliability of our method are reduced for larger communities as is expected in nonlinear dynamical systems with redundant and compensatory parameters. In our initial setup, an interaction coefficient was generated for every bacterium–metabolite pair. However, in both synthetic and natural communities, not every metabolite is produced or consumed by every bacterium, i.e. the bipartite network of bacterium–metabolite interactions is not fully connected. In our simulations, we observed that as community size increased, posterior distributions for interactions between bacteria and consumed metabolites concentrated near zero. This suggests that certain metabolites do not contribute to the growth of specific bacteria and could be omitted in simulated communities.

To investigate whether reducing the number of non-zero interaction coefficients improved model accuracy, we discarded interaction coefficients with absolute values below 0.2 and 0.5 in the large community, eliminating 38 (19%) and 114 (57%) interaction coefficients, respectively. Using the same inference approach, model diagnostics for both sparser communities showed no divergent transitions, with Rhat values close to one and large n_eff (Figs S9A and B and S10A and B). Comparisons of posterior distributions (Figs S9C and S10C), posterior predictive checks (Figs S9D and S10D), and comparison of assigned interaction coefficients with corresponding posterior means (Figs S9E and S10E) showed that the posteriors became more concentrated, with clearer modes moving away from zero as the number of nonzero interaction coefficients decreased. Credible intervals narrowed substantially in the sparser communities (Fig. 4C), and the MAE between the assigned interaction coefficients and corresponding posterior means also declined (Fig. 4D). These results indicate that Bayesian inference can identify interaction structures in large communities, provided that the connectance of the microbial network (i.e. the number of nonzero interaction coefficients) is sufficiently low.

## Discussion

A lack of theoretical foundation is hampering our understanding of the structure and function of microbial communities and consequently limits the development of novel microbiota-targeted interventions to improve the health of macro-ecosystems. Central to this problem is to infer and quantify microbial interactions and to develop predictive tools for community dynamics. To this end, we purposefully adopted an *in silico* approach where we treated the structure and parameter values of a simulated microbial community as the ground truth. This allowed us to assess the performance of our proposed Bayesian inference framework under controlled settings, without the interference of experimental noise and biological complexity, as is observed in real-world microbial communities. Within this context, our framework was able to accurately and reliably quantify interactions between bacteria and metabolites in simulated microbial communities. In small communities, we have shown that with noisy data due to observation errors, increasing the number of observations improves the accuracy of parameter estimation. In larger communities, our approach remains viable provided that the interaction network is relatively sparse. Overall, this framework provides a way to characterize microbial communities and lays the foundation for revealing the interaction network of real-world microbial communities to ultimately design microbiota-targeted interventions to support macro-ecosystem health.

### Incorporating biological realism with diverse growth forms and metabolite dynamics

A wide variety of quantitative methods have been used to capture microbial interactions, including Lotka–Volterra type models [25], correlation-based approaches [26], consumer-resource models [18] and agent-based models [27]. Modelling microbial interactions with these quantitative methods facilitates our understanding of the structure, dynamics, and functional properties of microbial networks [28]. Our generative model quantifies interactions using a set of coupled ODEs that describe the dynamics of consuming and excreting metabolites in a bacterial community. The growth of each bacterial species is described by the sum of all consumed metabolites multiplied by their corresponding interaction coefficients, thereby representing substitutable and additive bacterial growth dynamics commonly employed in consumer-resource models of microbial communities [18, 29]. This type of growth dynamics has been shown to predict microbial composition at the family level with high accuracy in an experimental setup [30]. Nonetheless, for specific systems or metabolic environments, more intricate forms of the per-capita growth function ${g}_j\left(\boldsymbol{M}\right)$ may be required. For example, even under the assumption of substitutable metabolites, per-capita growth ${g}_j\left(\boldsymbol{M}\right)$ saturates when metabolite concentrations are high. Because uptake and conversion rates are physiologically constrained by transporter and enzyme capacities, growth dynamics are better described by Monod kinetics: ${g}_j\left(\boldsymbol{M}\right)={c}_0\sum_{i\in \left\{M\right\}}{c}_{i,j}^{+}{M}_i/\left(1+{M}_i/{K}_i\right)$, with ${K}_i$ the half-saturation constant [31, 32]. The per-capita growth can also be limited by the scarcest metabolite following Liebig’s minimum principle, ${g}_j\left(\boldsymbol{M}\right)={c}_0\underset{i}{\min }{c}_{i,j}^{+}{M}_i$, or be colimited by multiple metabolites [33], ${g}_j\left(\boldsymbol{M}\right)={c}_0{\prod}_i{c}_{i,j}^{+}{M}_i/\left(1+{M}_i/{K}_i\right)$. Besides substitutable, some metabolites such as toxins can inhibit growth, ${g}_j\left(\boldsymbol{M}\right)={c}_0\sum_{i\in \left\{M\right\}}{c}_{i,j}^{+}{M}_i-\sum_{l\in \left\{\mathrm{inh}\right\}}{\gamma}_{l,j}{M}_l$, with $\left\{\mathrm{inh}\right\}$ the set of inhibitory metabolites and ${\gamma}_{l,j}$ the strength of inhibition of metabolite $l$ on bacterium $j$. More complex growth forms can also be considered, e.g. bacterium $j$ does not uptake a metabolite for growth until the concentration has reached a threshold [34]. All these variants of ${g}_j\left(\boldsymbol{M}\right)$ can be implemented in Eq. (1), depending on the research question or specific system of interest.

Prior knowledge of the system under study can be incorporated to add biological realism to metabolite dynamics. Although bacteria can interact directly via contact-dependent inhibition systems or type VI secretion systems [35], these interactions are not explicitly modelled in the present work but could be considered in future extensions. We focus solely on indirect interactions mediated through a shared metabolic environment. Here, all bacteria consumed the same seven metabolites and produced the same three metabolites, which is not representative of real-world communities, preventing the occurrence of cross-feeding interactions. However, by adjusting the sign of specific interaction coefficients, cross-feeding interactions can be incorporated into our model without the need for altering the dynamic model. For example, in a simple two-species community, *Bifidobacterium adolescentis* produces ethanol and acetate, while *Faecalibacterium prausnitzii* consumes acetate and produces butyrate [36]. In this case, the sign of the interaction between acetate and *B. adolescentis*, as well as acetate and *F. prausnitzii*, can be fixed to negative and positive values, respectively. Additionally, the interaction coefficient between *F. prausnitzii* and ethanol, and between *B. adolescentis* and butyrate, can be set to zero, which is particularly valuable, as constraining more entries of the interaction matrix to zero improves estimation for the remaining coefficients (Fig. 4C and D).

Overflow metabolism, where a bacterium produces and secretes a metabolite that is later taken up again, can be modelled by replacing the linear metabolite dependence in Eq. (2) with a Hill-type function of the form ${M}_i^n/\left({K}_i^n+{M}_i^n\right)$, whereby ${K}_i$ depicts the half saturation constant for metabolite $i$ and $n$ depicts the Hill coefficient, which determines the steepness of the switch [37]. Genome-scale metabolic models (GEMs) are useful tools to obtain such prior knowledge as these models are constructed from the genomic sequences and consist of experimentally validated or predicted biochemical reactions [38]. While GEMs for well-studied bacteria and communities (e.g. the human gut) are generally accurate and reliable, those for rare or poorly studied bacteria often contain erroneous or incomplete biochemical reactions [39]. In our simulations, the carrying capacity, metabolite conversion coefficients and death rates were held constant. These parameters can also be adjusted to better reflect the community under study or estimated using the same Bayesian inference framework by assigning appropriate priors, though increasing model complexity inevitably raises computational demands.

### Bayesian inference with informative priors

Beyond adjusting specific parameters, such as metabolite conversion rates, bacterial growth forms, or interaction coefficients, Bayesian statistical methods offer an inherent way to integrate biological realism. Model assumptions are made explicit through the choice of likelihoods and priors. Estimation accuracy can be improved by optimizing prior distributions or embedding prior selection in a reinforcement learning framework, where updated posteriors serve as priors as new data become available. In our study, posterior distributions of interaction coefficients were derived using broad uniform priors; narrowing these intervals or using alternative priors may better reflect biological reality and improve inference [40]. For the likelihood function, we used a lognormal distribution, constraining outcomes to non-negative, skewed distributions with multiplicative variability. This fits well with microbial systems: since metabolite concentrations and bacterial counts cannot be negative, most microbial communities follow highly skewed abundance distributions [41], and small differences in enzyme expression and catalysis can compound over time, affecting abundance distributions of bacterial taxa [42]. Environmental perturbations can shift species abundance patterns and alter interaction networks [43] as observed when comparing bacteria–metabolite networks from healthy versus type-2 diabetic gut microbiota [44], potentially requiring alternative likelihood choices.

Lastly, large-scale microbiota datasets contain many rare bacteria or metabolites, resulting in an excess number of zeros in the data, which can affect model performance [45]. Our model maintained accuracy, but uncertainty was increased under zero-inflated conditions. Appropriate likelihood choice can mitigate the effects of zero-inflated data. For example, zero inflation was modelled using a hierarchical Bayesian model with a likelihood that followed a Poisson-log-normal distribution containing a parameter describing the probability of zero inflation [46]. Accounting for zero inflation improved model performance when analysing gene abundance in metagenomic datasets obtained from the human gut microbiota [46], further emphasizing the importance of model selection for the system under study.

Finally, Bayesian inference regularizes parameter estimation due to the use of priors, which reduces overfitting even with small sample sizes or relatively few measurements [17]. While additional observations did improve accuracy of parameter estimation in our approach, the effect was modest (Fig. 3). Thus, Bayesian methods are particularly suitable for studying dynamic microbial systems, where sampling is often constrained by costs and logistics [47, 48]. Moreover, Bayesian methods explicitly quantify uncertainty, accommodating both stochasticity and experimental noise. Posterior distributions can be propagated to generate predictive distributions of microbial dynamics or metabolite trajectories, offering a direct means to assess responses to perturbations and to design microbiota-targeted interventions.

### Concluding remarks

The proposed Bayesian inference framework provides an accurate and reliable means of estimating bacteria–metabolite interactions in simulated microbial networks. The generative model offers a flexible backbone for representing diverse microbial communities, while its numerical implementation and Bayesian inference enable the quantification of network structures from empirical bacterial and metabolic data. When applied to real-world microbial communities, this approach can serve as a means to simulate the responses of microbiota-targeted interventions *in silico* by altering system properties such as network connectance, interaction strengths and species composition. In doing so, it can help identify possible intervention targets, including probiotics, prebiotics, and dietary alterations. Our combined approach may guide the development of microbiota-targeted interventions to improve the health of macro-ecosystems, including the human gut, by establishing a theoretical foundation for future studies.

## Supplementary Material

ycag066_Supplementary_files_Jansma_etal_revised_clean

## Data Availability

All code used in this study is publicly available at: https://github.com/JackJansma/Bayesian-inference-of-bacteria-metabolite-interactions. This includes R scripts for: (i) simulating bacteria–metabolite dynamics using ODE models, (ii) performing Bayesian parameter inference, and (iii) processing and visualizing the results. The simulations and analyses can be fully reproduced using the provided scripts, with the specified random seed ensuring reproducibility. The repository also includes documentation and information about required R packages and software dependencies. The generated data regarding all simulated communities and all parameter estimations using the Bayesian inference framework are publicly available at: https://doi.org/10.5281/zenodo.17078524

## References

[1] Sessitsch A, Wakelin S, Schloter M et al. Microbiome interconnectedness throughout environments with major consequences for healthy people and a healthy planet. *Microbiol Mol Biol Rev* 2023;87. PubMed PMID: 37367231:e0021222. 10.1128/mmbr.00212-22 37367231 PMC10521359

[2] Lin CH, Chen CC, Chiang HL et al. Altered gut microbiota and inflammatory cytokine responses in patients with Parkinson’s disease. *J Neuroinflammation* 2019;16:129. 10.1186/S12974-019-1528-Y 31248424 PMC6598278

[3] Wang X, Yuan W, Yang C et al. Emerging role of gut microbiota in autoimmune diseases. *Front Immunol* 2024;15:1365554. PubMed PMID: 38765017. 10.3389/fimmu.2024.1365554 38765017 PMC11099291

[4] Liu YY . Controlling the human microbiome. *Cell Syst* 2023;14:135–59. 10.1016/J.CELS.2022.12.010 36796332 PMC9942095

[5] Hugerth LW, Andersson AF. Analysing microbial community composition through amplicon sequencing: from sampling to hypothesis testing. *Front Microbiol* 8:1561. Available from: https://pmc.ncbi.nlm.nih.gov/articles/PMC5591341/. 10.3389/fmicb.2017.01561 PMC5591341 28928718

[6] Rath CM, Dorrestein PC. The bacterial chemical repertoire mediates metabolic exchange within gut microbiomes. *Curr Opin Microbiol* 2011;15:147–54. 10.1016/J.MIB.2011.12.009 22209085 PMC4112563

[7] Midani FS, David LA. Tracking defined microbial communities by multicolor flow cytometry reveals tradeoffs between productivity and diversity. *Front Microbiol* 13:910390. Available from: https://pmc.ncbi.nlm.nih.gov/articles/PMC9849913/. 10.3389/fmicb.2022.910390 PMC9849913 36687598

[8] Wishart DS, Cheng LL, Copié V et al. NMR and metabolomics—a roadmap for the future. *Metabolites* 2022;12:678. 10.3390/METABO12080678 35893244 PMC9394421

[9] Noecker C, Eng A, Muller E et al. MIMOSA2: a metabolic network-based tool for inferring mechanism-supported relationships in microbiome-metabolome data. *Bioinformatics* 2022;38:1615–23. 10.1093/BIOINFORMATICS/BTAC003 34999748 PMC8896604

[10] Morton JT, Aksenov AA, Nothias LF et al. Learning accurate representations of microbe-metabolite interactions. *Nat Methods* 2019;16:1306–14. 10.1038/S41592-019-0616-3 31686038 PMC6884698

[11] Cobo-López S, Gupta VK, Sung J et al. Stochastic block models reveal a robust nested pattern in healthy human gut microbiomes. *PNAS Nexus* 2022;1:pgac055. 10.1093/PNASNEXUS/PGAC055 PubMed PMID: 36741465. 36741465 PMC9896942

[12] Gupta G, Ndiaye A, Filteau M. Leveraging experimental strategies to capture different dimensions of microbial interactions. *Front Microbiol* 2021;27:700752. 10.3389/FMICB.2021.700752/XML PMC8503676 34646243

[13] Schlomann BH, Parthasarathy R. Timescales of gut microbiome dynamics. *Curr Opin Microbiol* 2019;50:56–63. 10.1016/J.MIB.2019.09.011 31689582 PMC6899164

[14] Ruigrok RAAA, Weersma RK, Vich VA. The emerging role of the small intestinal microbiota in human health and disease. *Gut Microbes* 2023;15:2201155. 10.1080/19490976.2023.2201155 37074215 PMC10120449

[15] Wang H, Cao J. pCODE: estimating parameters of ODE models. *R J* 14:291–304 Available from: https://pcode.shinyapps.io/pcode/.

[16] Cockayne E, Van De Walle A. Building effective models from sparse but precise data: application to an alloy cluster expansion model. *Phys Rev B Condens Matter Mater Phys* 81:012104. https://journals.aps.org/prb/abstract/10.1103/PhysRevB.81.012104

[17] McElreath R , editor. Statistical Rethinking: A Bayesian Course with Examples in R and Stan. 2nd ed. Boca Raton, FL: CRC Press; 2020. 10.1201/9780429029608

[18] Butler S, O’Dwyer JP. Stability criteria for complex microbial communities. *Nat Commun* 2018;9:1–10. PubMed PMID: 30061657. 10.1038/s41467-018-05308-z 30061657 PMC6065391

[19] Abreu CI, Friedman J, Andersen Woltz VL et al. Mortality causes universal changes in microbial community composition. *Nat Commun* 2019;10:1–9. 10.1038/s41467-019-09925-0 31073166 PMC6509412

[20] Cylke A, Serbanescu D, Banerjee S. Energy allocation theory for bacterial growth control in and out of steady state. Proceedings of the Royal Society a: mathematical. *Phys Eng Sci* 2024;480:2024.01.09.574890. 10.1098/RSPA.2024.0219

[21] Jansma J, Chatziioannou AC, Castricum K et al. Metabolic network construction reveals probiotic-specific alterations in the metabolic activity of a synthetic small intestinal community. *mSystems* 2023;8:e00332-23. 10.1128/msystems.00332-23 PMC10654062 37668401

[22] Guo J, Gabry J, Goodrich B et al. R Interface to Stan [R package rstan version 2.32.7]. *CRAN: Contributed Packages* 2025. 10.32614/CRAN.PACKAGE.RSTAN

[23] Van Leeuwen PT, Brul S, Zhang J et al. Synthetic microbial communities (SynComs) of the human gut: design, assembly, and applications. *FEMS Microbiol Rev* 2023;47:fuad012. 10.1093/FEMSRE/FUAD012 PMC10062696 36931888

[24] Berg G, Rybakova D, Fischer D et al. Microbiome definition re-visited: old concepts and new challenges. *Microbiome* 2020;8:1–22. 10.1186/s40168-020-00875-0 32605663 PMC7329523

[25] Camacho-Mateu J, Lampo A, Sireci M et al. Sparse species interactions reproduce abundance correlation patterns in microbial communities. *Proc Natl Acad Sci USA* 2024;121:e2309575121. 10.1073/pnas.2309575121 38266051 PMC10853627

[26] Kishore D, Birzu G, Hu Z et al. Inferring microbial co-occurrence networks from amplicon data: a systematic evaluation. *mSystems* 2023;8:e00422-23 10.1128/msystems.00961-22 PMC10469762 37338270

[27] Valiei A, Dickson A, Aminian-Dehkordi J et al. Metabolic interactions shape emergent biofilm structures in a conceptual model of gut mucosal bacterial communities. *NPJ Biofilms Microbiomes* 2024;10:99. 10.1038/S41522-024-00572-Y 39358363 PMC11447261

[28] Srinivasan S, Jnana A, Murali TS. Modeling microbial community networks: methods and tools for studying microbial interactions. *Microb Ecol* 2024;87:1–16. 10.1007/S00248-024-02370-7 PMC11001700 38587642

[29] Li Z, Liu B, Li SHJ et al. Modeling microbial metabolic trade-offs in a chemostat. *PLoS Comput Biol* 2020;16:e1008156. 10.1371/JOURNAL.PCBI.1008156 32857772 PMC7482850

[30] Estrela S, Sanchez-Gorostiaga A, Vila JCC et al. Nutrient dominance governs the assembly of microbial communities in mixed nutrient environments. *elife* 2021;10:e65948. 10.7554/ELIFE.65948 33877964 PMC8057819

[31] Venkatesh KV, Doshi P, Rengaswamy R. An optimal strategy to model microbial growth in a multiple substrate environment. *Biotechnol Bioeng* 56:635–44 Available from: https:///doi/pdf/10.1002/%28SICI%291097-0290%2819971220%2956%3A6%3C635%3A%3AAID-BIT6%3E3.0.CO%3B2-O. 10.1002/(SICI)1097-0290(19971220)56:6<635::AID-BIT6>3.0.CO;2-O 18642335

[32] Liu Y . Overview of some theoretical approaches for derivation of the Monod equation. *Appl Microbiol Biotechnol* 2007;73:1241–50. 10.1007/s00253-006-0717-7 17119956

[33] Delattre H, Desmond-Le Quéméner E, Duquennoi C et al. Consistent microbial dynamics and functional community patterns derived from first principles. *ISME J* 2018;13:263–76. 10.1038/S41396-018-0272-0 30194430 PMC6331576

[34] Casey JR, Follows MJ. A steady-state model of microbial acclimation to substrate limitation. *PLoS Comput Biol* 2020;16:e1008140. 10.1371/JOURNAL.PCBI.1008140 32845915 PMC7478835

[35] Garcia EC . Contact-dependent interbacterial toxins deliver a message. *Curr Opin Microbiol* 2018;42:40–6. 10.1016/j.mib.2017.09.011 29078204 PMC5899628

[36] El-Semman IE, Karlsson FH, Shoaie S et al. Genome-scale metabolic reconstructions of Bifidobacterium adolescentis L2-32 and Faecalibacterium prausnitzii A2-165 and their interaction. *BMC Syst Biol* 2014;8:1–11. 10.1186/1752-0509-8-41 24708835 PMC4108055

[37] Basan M, Hui S, Okano H et al. Overflow metabolism in E. coli results from efficient proteome allocation. *Nature* 2015;528:99–104. 10.1038/NATURE15765 26632588 PMC4843128

[38] Heinken A, Hertel J, Acharya G et al. Genome-scale metabolic reconstruction of 7,302 human microorganisms for personalized medicine. *Nat Biotechnol* 2023;41:1320–31. 10.1038/s41587-022-01628-0 36658342 PMC10497413

[39] Bernstein DB, Akkas B, Price MN et al. Evaluating E. coli genome-scale metabolic model accuracy with high-throughput mutant fitness data. *Mol Syst Biol* 2023;19:e11566. 10.15252/msb.202311566 PMC10698504 37888487

[40] Schulz S, Zondervan-Zwijnenburg M, Nelemans SA et al. Systematically defined informative priors in Bayesian estimation: an empirical application on the transmission of internalizing symptoms through mother-adolescent interaction behavior. *Front Psychol* 12:620802 Available from: www.frontiersin.org. 10.3389/fpsyg.2021.620802 PMC8024698 33841250

[41] Bickel S, Or D. The chosen few—variations in common and rare soil bacteria across biomes. *ISME J* 2021;15:3315–25. 10.1038/S41396-021-00981-3 34035442 PMC8528968

[42] Tonn MK, Thomas P, Barahona M et al. Stochastic modelling reveals mechanisms of metabolic heterogeneity. *Commun Biol* 2019;2:1–9. PubMed PMID: 30911683. 10.1038/s42003-019-0347-0 30911683 PMC6428880

[43] Dai T, Wen D, Bates CT et al. Nutrient supply controls the linkage between species abundance and ecological interactions in marine bacterial communities. *Nat Commun* 2022;13:1–9. 10.1038/s41467-021-27857-6 35013303 PMC8748817

[44] Sung J, Kim S, Cabatbat JJT et al. Global metabolic interaction network of the human gut microbiota for context-specific community-scale analysis. *Nat Commun* 2017;8:1–12. 10.1038/ncomms15393 28585563 PMC5467172

[45] Wu H, Guzior DV, Martin C et al. Longitudinal analyses of infants’ microbiome and metabolome reveal microbes and metabolites with seemingly coordinated dynamics. *Commun Biol* 2024;7:1506. 10.1038/S42003-024-07015-6 39543263 PMC11564710

[46] Jonsson V, Österlund T, Nerman O et al. Modelling of zero-inflation improves inference of metagenomic gene count data. *Stat Methods Med Res* 2019;28:3712–28. 10.1177/0962280218811354 30474490

[47] Hajihosseini M, Amini P, Saidi-Mehrabad A et al. Infants’ gut microbiome data: a Bayesian marginal zero-inflated negative binomial regression model for multivariate analyses of count data. *Comput Struct Biotechnol J* 2023;21:1621–9. 10.1016/J.CSBJ.2023.02.027 36860341 PMC9969297

[48] He J, Wang W, Huang M et al. Bayesian inference under small sample sizes using general noninformative priors. *Mathematics* 2021;9:2810. 10.3390/MATH9212810

